# ‘Small cost to pay for peace of mind’: Women's experiences with non‐invasive prenatal testing

**DOI:** 10.1111/ajo.12945

**Published:** 2019-02-06

**Authors:** Hilary Bowman‐Smart, Julian Savulescu, Cara Mand, Christopher Gyngell, Mark D. Pertile, Sharon Lewis, Martin B. Delatycki

**Affiliations:** ^1^ Murdoch Children's Research Institute Melbourne Victoria Australia; ^2^ Monash University Melbourne Victoria Australia; ^3^ Uehiro Centre for Practical Ethics University of Oxford Oxford UK; ^4^ Department of Paediatrics University of Melbourne Melbourne Victoria Australia; ^5^ Victorian Clinical Genetics Services Melbourne Victoria Australia

**Keywords:** genetic services, genetic testing, pregnancy, prenatal diagnosis, prenatal screening

## Abstract

**Background:**

Non‐invasive prenatal testing (NIPT) has been available in Australia on a user‐pays basis since 2012. Since its introduction, it has grown in popularity as a screening method for fetal aneuploidy and may become publicly funded.

**Aims:**

To assess the motivations and experiences of women who have undergone NIPT in a user‐pays system in Australia.

**Materials and methods:**

One thousand women who had undergone NIPT through the Victorian Clinical Genetics Services in Melbourne, Australia were contacted and asked to complete a mixed‐methods survey. The number of eligible responses received was 235. Quantitative data analysis was performed in STATA IC 15.1, and qualitative data were examined using content analysis.

**Results:**

Women reported generally positive experiences with NIPT and 95% of respondents indicated they would undergo NIPT in a future pregnancy. Most respondents received a low‐risk result, with 2.2% receiving a high‐risk result. Respondents viewed NIPT favourably compared to invasive testing and cited reassurance as a key reason they sought it. However, a small minority of women reported negative experiences with the testing process. Women were also supportive of NIPT becoming publicly funded, with 93% of respondents indicating support. Pre‐ and post‐test counselling were identified as possible areas for improvement to ensure informed consent.

**Conclusion:**

In support of the existing literature, these results indicate that Australian women generally report positive experiences with NIPT. As NIPT becomes more common, with possible integration into public healthcare, further qualitative research would be valuable to examine the motivations and experiences of women undergoing NIPT.

## Introduction

Prenatal tests commonly screen for chromosomal conditions such as trisomy 21 (Down syndrome). In Victoria, yearly uptake for the most common publicly funded screening method, combined first trimester screening (CFTS), is consistently more than 80% of pregnancies.^1^ A newer screening method is non‐invasive prenatal testing (NIPT). NIPT is based on cell‐free fetal DNA (cffDNA) in the maternal bloodstream. A blood sample is taken from the mother, and cffDNA is analysed to produce a probability of the fetus having a chromosomal condition.^2^ However, NIPT is not diagnostic for the trisomy disorders^2^ and any high‐risk result should be confirmed with invasive diagnostic testing, such as amniocentesis.

Non‐invasive prenatal testing has many benefits over CFTS. NIPT is more accurate, with a very high sensitivity and specificity for trisomy 21 (>99%).^2^ NIPT outperforms CFTS in both low‐ and high‐risk populations.^3^ NIPT can also be performed earlier in the pregnancy, usually from 10 weeks gestation.^4^

Non‐invasive prenatal testing became available on a user‐pays basis to Victorian women in 2012, and this was associated with a 22.9% reduction in invasive testing,^5^ due to the much lower false‐positive rate of NIPT compared to CFTS.^1^ Availability of NIPT has had similar impacts on number of referrals for diagnostic tests in other healthcare systems.^6^

Non‐invasive prenatal testing in Australia currently costs approximately AUD$450.^7^ Although this is less expensive than many other countries, cost remains the major barrier to widespread uptake.^8^ NIPT is being implemented in other public healthcare systems such as the NHS in the United Kingdom.^9^ Critical questions remain for possible implementation of NIPT in Australian public healthcare, such as the criteria for access to screening.^10^

Women have reported positive experiences with NIPT, emphasising accuracy, ease and safety.^11^, ^12^ These aspects of NIPT have also emerged as important motivators for women to use it; other reasons include seeking reassurance and how early in the pregnancy NIPT can be done.^13^ Concerns have been raised about the impact that routinisation of NIPT may have on informed choice; however, previous research has found high levels of informed choice among women who have undergone NIPT.^14^, ^15^

There is limited literature assessing experiences with NIPT in the Australian context.^16^, ^17^ In this study, we aimed to examine the experiences and motivations of women who had undergone NIPT through the Victorian Clinical Genetics Services (VCGS) in Victoria, Australia.

## Materials and Methods

One thousand women who had undergone the *percept* ^™^ NIPT through VCGS were selected using systematic sampling (every fifth name from a selected point) from a list of 14 680 referrals in 2016. They were contacted by post in two rounds of 500 (October 2017 and February 2018) and asked to complete a survey, either online in REDCap or through hard copy. Those under the age of 18 were excluded. Respondents were advised that by commencing the survey they were consenting to use of their response data.

The survey was developed in conjunction with professionals with relevant expertise in clinical genetics, bioethics, moral psychology and public health, with feedback from mothers outside the research group. Respondents were asked about motivations for undergoing NIPT, experiences with the process, and levels of satisfaction. These included questions about how informed they felt and the results of their test. Quantitative data (85 questions) were collected as categorical variables, including five‐point Likert scales. Qualitative data were collected in open‐ended questions (four). Demographic data were also sought. A number of questions assessing women's attitudes to future uses of NIPT were included and will be published elsewhere. The survey can be found in supplementary material.

Data were exported for analysis into STATA IC 15.1 (StataCorp, College Station, TX, USA) and NVivo 12 (QSR International Pty Ltd, Melbourne, Victoria, Australia). Frequencies were generated and are presented as percentages, along with numbers of cases. Qualitative data were assessed by two researchers (HBS and CM) independently in NVivo using content analysis. Common themes addressing experiences with NIPT were identified and coded, and the two sets of codes were integrated with discussion. Quotes from respondents are presented with their number, age group, and NIPT results in brackets (eg #47, 31‐35, no increased risk). The quotes are not necessarily representative of the sample, but provide insights into the most important themes identified during analysis.

The study was conducted with the approval of the Royal Children's Hospital Human Research Ethics Committee (37154C) and the Monash University Human Ethics Committee (10576).

## Results

The number of women who responded to the survey was 237. Two were excluded as they did not specify age, and therefore were ineligible to participate. Demographic features are seen in Table 1.

**Table 1:** Demographic features of the cohort

	Participants (*n*)	Percentage
Age (*n *=* *235)		
18–25	0	0%
26–30	23	9.8%
31–35	93	39.6%
36–40	79	33.6%
41+	40	17%
Highest level of education (*n *=* *225)		
Primary school	0	0%
Secondary school	19	8.4%
Technical or trade certificate	27	12%
Bachelor's degree	106	47.1%
Postgraduate qualification (e.g. Masters, PhD)	73	32.4%
Number of children (*n *=* *227)		
1	126	55.5%
2	77	33.9%
3	21	9.3%
4	2	0.9%
5+	1	0.4%
Further children planned (*n *=* *223)		
Yes	88	39.5%
No	71	31.8%
Currently pregnant	13	5.8%
Unsure	51	22.9%
Marital status (*n *=* *225)		
Single	5	2.2%
Partnered	39	17.3%
Married	180	80%
Divorced	1	0.4%
Household income (*n *=* *219)		
Less than $25 000	1	0.5%
$25 000–$49 999	5	2.3%
$50 000–$69 999	6	2.7%
$70 000–$99 999	27	12.3%
$100 000–$129 999	46	21%
$130 000–$149 999	35	16%
More than $150 000	99	45.2%

### Reasons for undergoing NIPT

**Figure 1:**
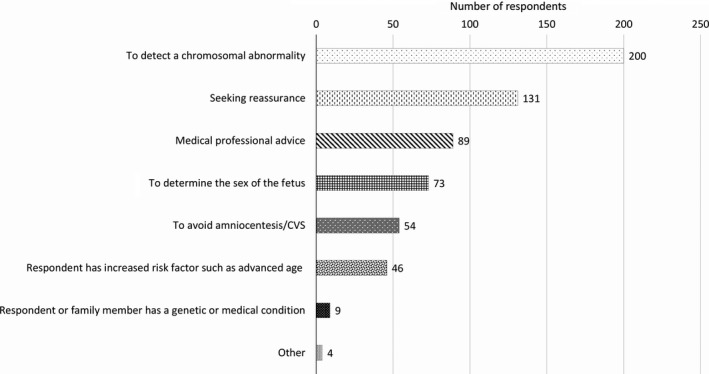
Reasons for undergoing non‐invasive prenatal testing (*n* = 233). Respondents could select more than one option. (CVS: chorionic villi sampling)

Many respondents (*n *=* *200, 85.8%) indicated that detecting chromosomal abnormalities was a reason they underwent NIPT (Fig. 1). A minority of 31.3% (*n *=* *73) wanted to determine fetal sex (Fig. 1). Advice from a medical professional was also a prevalent response, with 38.2% (*n *=* *89) indicating this was a reason for undergoing testing (Fig. 1).

Seeking reassurance and ‘peace of mind’ emerged as important motivators for undergoing NIPT, with 56% (*n *=* *131) of respondents selecting it (Fig. 1). This was reflected in the open‐ended responses.

Having the NIPT results available provided much reassurance that we wouldn't have had otherwise and essentially assisted in our choice not to have an amniocentesis to further investigate. (#47, 31–35, no increased risk)

The NIPT testing brought me so much peace of mind…in my pregnancy…small cost to pay for peace of mind. (#26, 36–40, no increased risk)

Twenty‐three percent of women (*n *=* *54) were motivated to undergo NIPT to avoid invasive testing (Fig. 1). Respondent #43 (36–40, no increased risk) highlighted the ease and non‐invasiveness of NIPT.

In comparison [to amniocentesis], the NIPT test was a fair [sic] quicker procedure, obviously no different to a blood test, and far less stressful…I would highly recommend…to certainly undergo the NIPT test, rather than an amniocentesis.

### Service providers

**Figure 2:**
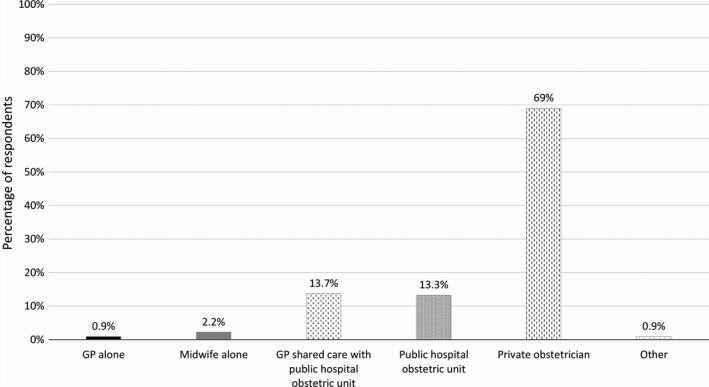
Primary treating professional (*n* = 226). (GP: general practitioner)

The primary treating professional was a private obstetrician in 69% (*n *=* *156) of cases, while 27.9% (*n *=* *63) indicated they were primarily treated by a general practitioner (*n *=* *2), public hospital obstetric unit (*n *=* *30), or combination thereof (*n *=* *31; Fig. 2). Only 2.2% (*n *=* *5) indicated that they were primarily treated by a midwife (Fig. 2).

### Attitudes toward funding

**Figure 3:**
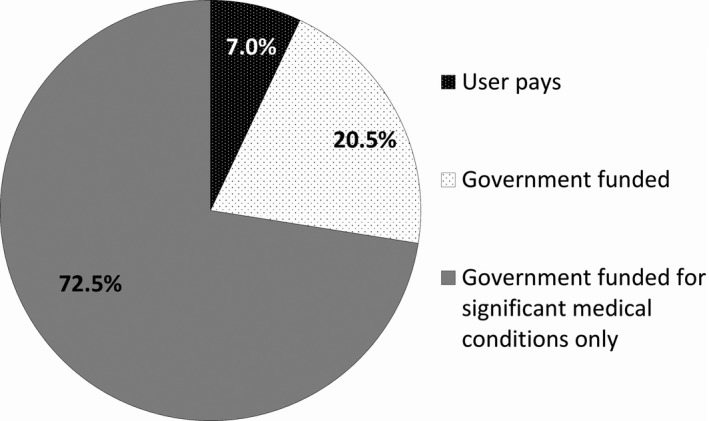
Attitudes towards how non‐invasive prenatal testing should be funded (*n *=* *229)

Respondents were favourable toward NIPT being integrated into public healthcare (Fig. 3). Respondents indicated an interest in ensuring public funding was directed to where it was perceived as ‘necessary’, excluding non‐medical traits.

More important that the testing is available and government subsidised than to have an expensive fancy test that provides results that really are inconsequential. (#166, 41 + , no increased risk)

### Test results

The majority of women received a low‐risk result (97.8%, *n *=* *227). Three women (1.3%) received a high‐risk result for a trisomy disorder, one woman (0.4%) received a high‐risk result for a sex chromosome aneuploidy (false positive), and one woman (0.4%) indicated a high‐risk result of ‘other’. Therefore, the proportion of women who received any high‐risk result was 2.2%, comparable to the overall number of high‐risk results from the *percept* test (2.1%, internal VCGS data).

The three women who received a high‐risk result for a trisomy disorder confirmed the diagnosis with invasive testing, and all three opted for termination of pregnancy.

### Experience with the NIPT process

Women's experiences with NIPT were generally positive (Table 2). Most respondents (*n *=* *218, 94%) felt they were provided with adequate information on the nature of the test and possible results. Most respondents (*n *=* *219, 95%) also indicated they would probably or definitely undergo NIPT again.

**Table 2:** Experiences with the non‐invasive prenatal testing (NIPT) process

	Definitely	Mostly/probably	Neutral/unsure	Mostly not/probably not	Definitely not
Do you feel you were provided with adequate information on the nature of the *percept*™ test and possible results? (*n *=* *232)	149 (64.2%)	69 (29.7%)	9 (3.9%)	4 (1.7%)	1 (0.4%)
Do you feel you were sufficiently informed of what the consequences of a positive result (eg for trisomy 21 (Down syndrome)) would be? (*n *=* *232)	79 (34.1%)	74 (31.9%)	36 (15.5%)	36 (15.5%)	7 (3%)
Do you feel you were provided with adequate information and counselling after you received the results of the *percept*™ test? (*n *=* *232)	110 (47.4%)	71 (30.6%)	41 (17.7%)	7 (3%)	3 (1.3%)
If you had another pregnancy, how likely would you be to undergo NIPT again? (*n *=* *231)	192 (83.1%)	27 (11.7%)	9 (3.9%)	0 (0%)	3 (1.3%)

Should I get pregnant again I will be 100% do this testing at the 11 week mark so that if it did come back that our child did have a condition that we would at least have options. (#26, 36–40, no increased risk)

Although most respondents (*n *=* *181, 78%) felt they were provided with adequate counselling after the receipt of results, a considerable minority were either neutral or had negative experiences (*n *=* *51, 22%). Similarly, 66% of respondents (*n *=* *153) felt they were sufficiently informed of what the consequences of a high‐risk result for aneuploidy might be.

While most respondents had a positive experience, some respondents explained further in open‐ended responses that they had negative or ambivalent experiences with the process.

Respondent #78 highlighted issues with informed consent and pre‐test counselling; she reported feeling pressured by other parties, including medical professionals and family.

I felt pressured into taking the NIPT test by my OB, GP and Husband…Before the NIPT test no‐one asked me if I would terminate should the baby have downs syndrome and…I decided that I would not terminate under any circumstances. I then took the test and paid the $500 to get everyone off my back! (#78, 36–40, no increased risk)

Respondent #132 received a high‐risk result but did not specify the condition, although in an open‐ended response she indicated the result was intermediate risk of ‘DS’ (trisomy 21). She highlighted concerns both with informed consent and anxiety surrounding the results. She did not undergo invasive testing after NIPT, and her child was not born with an aneuploidy. She felt that she was ‘mostly’ sufficiently informed as to the consequences of a high‐risk result, but she felt that she ‘definitely [did] not’ receive adequate counselling after the test. She expressed concerns about the accuracy of NIPT, referring to them as ‘*broken results*’ causing unnecessary anxiety.

The Nipt [sic] test unfortunately put an enormous amount of stress throughout my pregnancy…The results were intermediate/inconclusive and my risk was 1:5 chance of DS….I got the test done hastily as a first time mum I just did what ever [sic] was given to me not actually thinking about what I would do with the information…I won't ever get [NIPT] done again due to the consequences that follows after you receive not so good results (#132, 31‐35, indicated received unspecified high‐risk result)

However, of the other four respondents who received high‐risk results, all indicated that they were ‘mostly’ (*n *=* *1) or ‘definitely’ (*n *=* *3) adequately informed of the consequences of a high‐risk result. The responses were the same when asked about adequate counselling after the test. They all indicated they would ‘definitely’ undergo NIPT again.

## Discussion

The experience of women accessing NIPT in Victoria, Australia is largely positive. This corresponds with previous research finding high levels of satisfaction and that few women regret undergoing NIPT, regardless of the result.^12^ The high percentage of women who indicated they would undergo NIPT again supports this conclusion. The introduction of NIPT represents a positive development in prenatal testing, especially when contrasted with invasive procedures; a considerable minority viewed NIPT as a positive alternative to invasive testing. Seeking reassurance emerged as an important motivator, consistent with previous studies.^13^, ^18^ Most respondents who received a high‐risk result (4/5) indicated they would definitely undergo NIPT again.

The respondents in our study were generally highly educated, wealthy and in a stable relationship (see Table 1), indicating high socioeconomic status. This is consistent with a previous study that showed women receiving NIPT‐indicated diagnoses in Victoria are more advantaged than those receiving diagnoses from other methods such as CFTS.^19^ This is unsurprising considering NIPT's cost. These results suggest NIPT is less accessible to women of low socioeconomic status, which would change if the test becomes publicly funded.

The most common primary treating professional was a private obstetrician (69%, Fig. 2). A 2015 study of Australian and New Zealand medical professionals found there was no significant difference between those working in public and private care offering NIPT to high‐risk women, with cost remaining the main access barrier.^16^ It is plausible private professionals may offer NIPT more frequently to low‐risk women (a category covering most respondents) than public healthcare professionals. However, it is equally possible low‐risk women most likely to be interested in NIPT seek out private care. Further research into the understanding of health professionals about NIPT and screening options may shed further light on these results.

The mean gestational age at blood draw for the VCGS cohort capturing the respondents in this sample was 11.0 ± 1.9 weeks, with approximately 80% of the total cohort using NIPT as their primary screening test.^20^ Approximately half the respondents were under the age of 35 at the time of completing the survey. Therefore, demand from a low‐ to average‐risk population appears to be high. In addition to the VCGS data, an audit of Australian women who had undergone NIPT up until the end of 2013 found that 21% had no specific risk factor indicating need for a referral (eg advanced age or high‐risk CFTS result).^21^ A 2016 study of 5267 Australian women found nearly two‐thirds had used NIPT as a first‐line screen, usually under 11 weeks gestation.^22^ Analysis of over 900 000 worldwide *Harmony* NIPT tests (Ariosa Diagnostics, San Jose, CA, USA) suggest the demand from the low‐risk population for NIPT is increasing,^23^ which is supported by our results.

Most respondents were not supportive of the current user‐pays system and would prefer some form of government funding. NIPT is becoming integrated into several public healthcare systems, such as the NHS.^24^ It is possible that NIPT will become subsidised through the Australian Medicare system, with applications having been made.^25^ Our results suggest women who have undergone NIPT are in favour of this. However, the question of what screening model should be implemented remains. Should all women have access to publicly funded NIPT or only those at high risk? A health economic analysis must be performed to identify the most appropriate screening model.

The responses support the position that women felt they had given informed consent. However, while the responses generally suggest that most respondents had a positive experience and would use NIPT again, there is room to improve in the areas of pre‐ and post‐test counselling. In particular, women who consider NIPT should be fully aware of the possibility and consequences of a high‐risk result. Referring professionals should be educated on how to present information about the test to enhance patients’ understanding.

Respondent #43's comment that NIPT is ‘no different to a blood test’ is echoed by the statement that it is ‘just a blood test’ found throughout the literature.^9^, ^26^, ^27^ Due to the low risk and ease of testing, NIPT could come to be part of the ‘standard’ set of prenatal tests, with implications for informed consent. Both healthcare professionals and potential consumers have indicated a belief in a decreased need for written consent and time between pre‐test counselling and testing.^28^, ^29^ This approach may result in a ‘one‐stop’ appointment, where blood is drawn immediately after pre‐test counselling. Levels of informed consent, although remaining high, have decreased with the implementation of NIPT as part of routine prenatal care in the NHS compared to the study that evaluated screening models due to the decrease in counselling time.^30^

Similarly, adequate pre‐ and post‐test counselling is critical to informing the choices women make. Respondent #78, who received a low‐risk result, reported she felt pressured to undergo NIPT. Although this was not a majority experience, it highlights the ongoing need for open conversations around women's preferences to ensure that women do not feel pressured to make a particular choice. A sizeable minority (22%, Table 2) of respondents were not positive about post‐test counselling. Respondent #132's negative experience may be due to the uncertain nature of her result. Implications and possible reasons for her result may not have been adequately communicated, resulting in distress and a perception the results are ‘broken’. One negative aspect of NIPT that has been reported in previous studies has been the anxiety and confusion caused by waiting for results, the receipt of ambiguous results, and fears of inaccuracy,^11^, ^30^ more prevalent in women with lower medical literacy.^12^

This study had several limitations. It addressed women who had NIPT within the current context of its provision in Australia, which is user‐pays (at time of writing, costing AUD$449^7^). Therefore, respondents were generally of high socioeconomic status. Women who are of lower socioeconomic status or referred from the public healthcare system may have different experiences. It was also biased toward those who were proactive in responding to the survey; the response rate was relatively low. This may result in, for example, responses from those with particularly positive or negative experiences of NIPT. Very few respondents had a high‐risk result, and therefore the views may be generally representative only of those who had a low‐risk result.

The findings of this study suggest that overall, women have positive experiences with NIPT and support implementation into the Australian public healthcare system. Additional development of pre‐ and post‐test counselling was identified as an area of importance to ensure informed consent. The data from this study support further in‐depth qualitative research into the motivations and experiences of women who have undergone NIPT, particularly important as it becomes more common as a prenatal screening option.

## Funding

There was no specific funding for this study. Research conducted at the Murdoch Children's Research Institute was supported by the Victorian Government's Operational Infrastructure Support Program. This work was supported by the Wellcome Trust [203132].
